# Supplementary material for: Predictors of antenatal care services utilization by pregnant women in Afghanistan: Evidence from the Afghanistan Health Survey 2018
Source: PLoS One. 2024 Oct 2;19(10):e0309300. doi: 10.1371/journal.pone.0309300 (PMC11446418; doi:10.1371/journal.pone.0309300)
Supplement: Attachment — Submitted filename: Response Letter.docx [file pone.0309300.s002.docx]

Dear Editor,

We would like to thank the respected editor and the respected reviewers for their thoughtful evaluation of our manuscript (ID Number, **PONE-D-24-05385R2**) entitled ***“******Predictors of Antenatal Care Services Utilization by Pregnant Women in Afghanistan: Evidence from the Afghanistan Health Survey 2018”***. Please find our revised manuscript (with highlighted changes), which we believe is substantially strengthened now that we have incorporated reviewers’ recommendations.

**Editor Comments**

Thank you for addressing the initial comments provided by the reviewers. Based on the revised manuscript, the reviewers have requested that some minor comments still need to be addressed.

Response: Thank you so much. We thank you and the reviewers for the positive and constructive feedback. In our point-by-point responses below, we have indicated text changes that have been made and line numbers from the tracked version of the manuscript.

**Response to Reviewers’ comments**

**Response to Reviewer 1 comments**

1. In Abstract Results: Last para needs to be restructured.

Response: Thank you very much. We have revised the last paragraph. Please see lines 43-47 on Page 2.

2. L95: I think there's a typo ANC1. Please recheck.

Response: Thank you so much for your meticulous observation. We have made corrections. Please see lines 91-92 on Page 4.

3. Figure 1 and 2, can be combined in 1.

Response: Thank you for your feedback. We have merged both figures into one (Please see Figure 1).

4. Please weigh out the importance of keeping figure 3.

Response: Thank you so much for this comment. We want to keep figure 3 as it highlights timely vs. late ANC according to the ANC visits.

5. Figure 4 can better be visualised as a pie chart.

Response: Thank you so much for this comment. We presented it as pie chart. Please see now Figure 3.

6. L223: I think the word "only should be removed before 63.2%.

Response: Thank you so much. We have removed the term only. Please see line 227 on Page 13.

7. Authors must read carefully to make corrections for grammatical errors and sentence structuring.

Response: Thank you so much. All authors have proof-read the entire revised manuscript for grammatical errors and sentence structuring. We have made corrections in all sections.

**Response to Reviewer 2 comments**

I would like to thank the authors for their revisions and the manuscript looks better, but I would suggest a few pending issues which must be addressed before this manuscript can be considered for publication.

Response: Thank you so much for your constructive feedback. We have incorporated all your feedback in the revised version of our manuscript.

1. The language would still need some editing especially in the introduction and the discussion section and please avoid using terms like 'not surprisingly', 'important issue', 'pronounced', 'for instance'.

Response: Thank you so much. All authors have proof-read the entire revised manuscript for language correction. We have also avoided terms specifically noted by the esteemed reviewer.

2. Figure 1 and figure 2 should be merged.

Response: Thank you for this feedback. We have merged both figures into one (Please see Figure 1).

3. The discussion section is still lengthy and should be shortened.

Response: Thank you for this suggestion. We agree with you, and we have reduced the text in the discussed section where appropriate (Please see the discussion section).

We would like to thank once again the respected editor and esteemed reviewers for their constructive feedback and help, and we hope that the revised version of our manuscript will be deemed suitable for publication in this prestigious journal.
